# Right atrial myxoma after catheter ablation

**DOI:** 10.1007/s12574-025-00687-x

**Published:** 2025-04-06

**Authors:** Tomoya Hasegawa, Junya Tanabe, Koji Shimizu, Nobuhide Watanabe, Hiroyuki Yoshitomi, Kazuhiro Yamazaki, Kazuaki Tanabe

**Affiliations:** 1 Division of Cardiology, Faculty of Medicine, Shimane University, Izumo, Japan; 2 Department of Cardiovascular Surgery, Faculty of Medicine, Shimane University, Izumo, Japan

The female patient in her 70 s underwent radiofrequency ablation (RFA) for paroxysmal atrial fibrillation, including pulmonary vein isolation and linear ablation of the cavotricuspid isthmus. The procedure time for RFA was 3 h 51 min, including 28 min 47 s of fluoroscopy time. The patient has been on warfarin for anticoagulation; however, six months after RFA, the patient experienced cerebral hemorrhage and subsequently began dialysis. Since the sinus rhythm remained stable, warfarin treatment was discontinued.

One year after RFA, transthoracic echocardiography (TTE) revealed a previously undetected 17 × 11 mm mobile mass in the right atrium (Fig. 1A–C, videos 1 and 2). Suspecting a thrombus, warfarin was resumed. Nevertheless, the mass increased to 20 × 17 mm and 21 × 20 mm at 1.5 and 2 years after the RFA, respectively, prompting surgical resection (Fig. 1D, videos 3, 4). Cardiac magnetic resonance imaging showed low signal intensity on both T1-weighted and T2-weighted images.

**Fig. 1:**
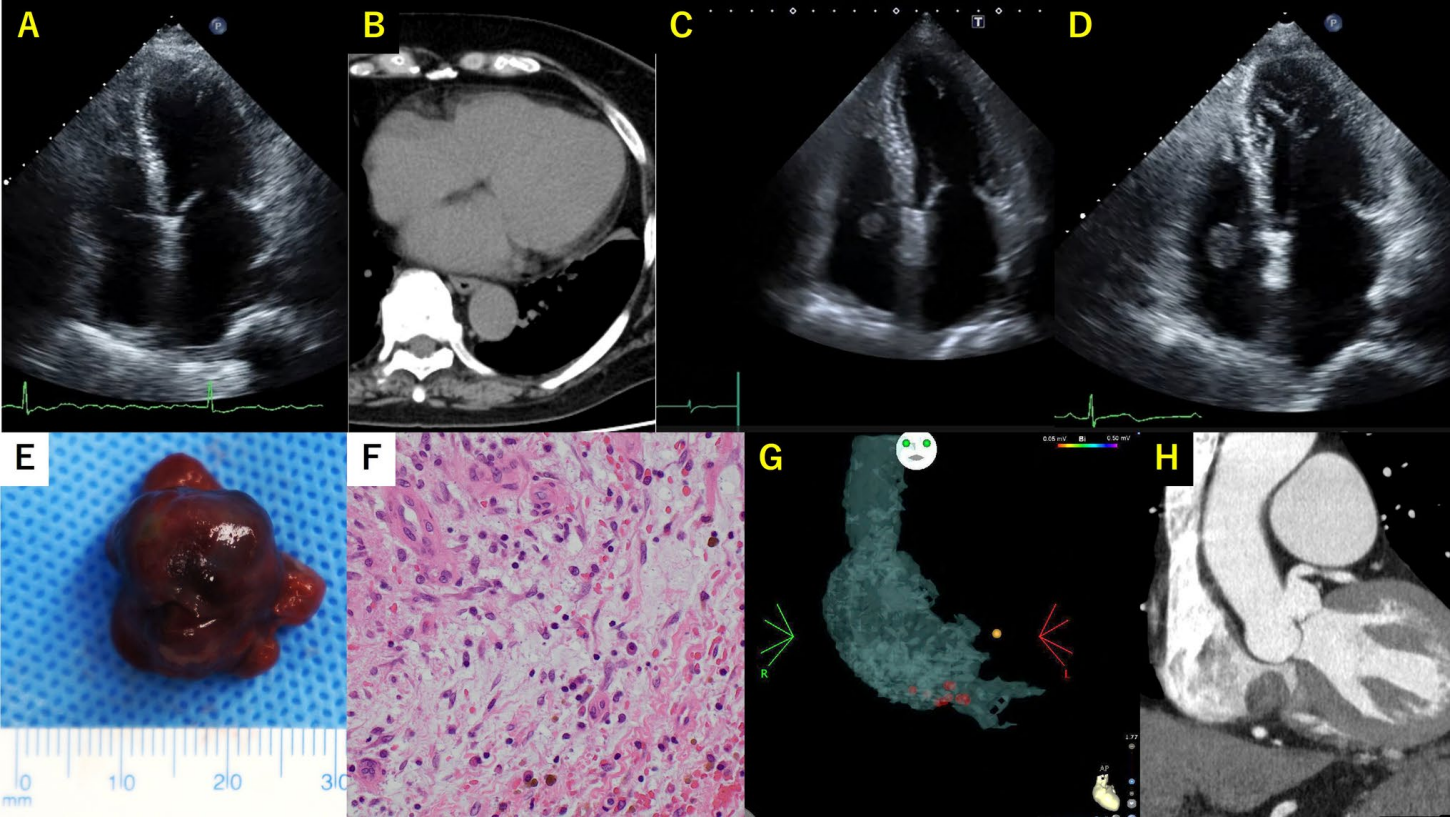
Panel **A**. Transthoracic echocardiogram (TTE) apical four-chamber view, performed before radiofrequency ablation (RFA), showed no mass in the right atrium. Panel **B**. Computed tomography (CT) horizontal section, performed before RFA, showed no mass in the right atrium. Panel **C**. TTE apical four-chamber view, performed one year after RFA, revealed a 17 mm × 11 mm mobile mass in the right atrium. Panel **D**. TTE apical four-chamber image performed two years after RFA, revealed a 21 mm × 20 mm mobile mass in the right atrium. Panel **E**. Surgically removed 20 mm x 20 mm sized mass. Panel **F**. Pathological findings of the excised mass (strongly magnified image). Panel **G** and **H**. Ablation sight at the cavotricuspid isthmus (G) compared with preoperative cardiac contrast-enhanced CT coronal section (H)

The surgically excised mass measured 20 × 20 mm, had a smooth surface, and was attached to the valvular annulus at the commissure between the septal and the posterior leaflets of the tricuspid valve in the coronary sinus orifice (Fig. 1E).

Histopathological examination revealed spindle-shaped cells proliferating within a myxoid and loose extracellular matrix background, which proliferating along with blood vessels, and cells with hemosiderin in the cytoplasm. Hemorrhage and lymphocytic infiltration were also confirmed, and a diagnosis of cardiac myxoma and intra-tumor bleeding was made (Fig. 1F). The postoperative recovery was uneventful, and the patient was discharged for 16 days postoperatively.

Cardiac myxomas occur in about 75% of left atrial and 20% of right atrial cases, mostly attaching to the atrial septum. In this patient, the myxoma extended from the coronary sinus orifice to the tricuspid valve annulus, making diagnosis and differentiation from thrombus challenging [1, 2]. Few reports exist on cardiac myxoma after RFA.

Prolonged fluoroscopy, tissue damage, and inflammation from RFA, along with heat energy, have been speculated to contribute to heart neoplasms, potentially playing a role in myxoma development. However, a direct causal relationship remains unclear [3, 4].

In this case, the attachment site of the myxoma coincided with the ablation site, suggesting a potential causal relationship with RFA (Fig. 1G, H). Although, no masses appeared in areas where atrial septal puncture or pulmonary vein isolation was performed.

## Supplementary Information

Below is the link to the electronic supplementary material.

Supplementary file1 Video 1. Transthoracic echocardiogram (TTE) apical four-chamber image before radiofrequency ablation (RFA) (MP4 6879 KB)

Supplementary file2 Video 2. TTE apical four-chamber image atoneyear after RFA (MP4 1790 KB)

Supplementary file3 Video 3. TTE apical four-chamber image at one and a halfyears after RFA (MP4 1204 KB)

Supplementary file4 Video 4. TTE apical four-chamber image at two years after RFA (MP4 5539 KB)

## Data Availability

Data sharing is not applicable to this article as no datasets were generated or analyzed during the current study.
